# Strength-Ductility Synergy in a Metastable β Titanium Alloy by Stress Induced Interfacial Twin Boundary ω Phase at Cryogenic Temperatures

**DOI:** 10.3390/ma13214732

**Published:** 2020-10-23

**Authors:** Yongkang Li, Zhibin Liao, Weidong Zhang, Zhenggang Wu, Canxu Zhou

**Affiliations:** 1 College of Materials Science and Engineering, Hunan University, Changsha 410082, China; lykang@hnu.edu.cn (Y.L.); zhibinliao@hnu.edu.cn (Z.L.); zwu9@hnu.edu.cn (Z.W.); 2 School of Materials Science and Engineering, Tsinghua University, Beijing 100084, China; canxuzhou@tsinghua.edu.cn

**Keywords:** titanium alloy, cryogenic mechanical properties, twinning, ω phase, martensitic transformation

## Abstract

A β titanium alloy is an excellent candidate for cryogenic applications. In this study, the deformation behavior of Ti-36Nb-2Ta-3Zr-0.35O with cold swaging was investigated at cryogenic temperatures to verify its practical application value. The microstructure after tensile tests was observed by transmission electron microscope in order to reveal the cryogenic deformation mechanism. The results show that the mechanical properties of this alloy have a strong temperature dependence: an increase in strength with a non-monotonic trend (first increase and then decrease) in elongation is found when the temperature decreases from 297 K to 77 K. At 200 K, a strength-ductility synergy is obtained and is mainly due to the occurrence of {211} <11> mechanical twinning accompanied with the ω plate located at the twin boundaries, which is the first time it is detected in titanium alloy at a cryogenic temperature. However, at 77 K, martensitic transformation (β phase to α phase) is induced by the tensile deformation, leading to the increase of strength with a massive sacrifice of elongation. These findings provide insights for understanding the deformation mechanisms and optimizing the mechanical properties of titanium alloys at a cryogenic temperature.

## 1. Introduction

Titanium alloys are being used more widely as the cryogenic engineering material due to their unique properties at cryogenic temperatures such as a high strength-to-weight ratio, good toughness, low expansion coefficient, and excellent corrosion resistance [1,2,3]. At present, the major titanium alloys used at cryogenic temperature include pure titanium, the α type, and α + β type titanium alloys. In addition, there are extensive research studies to investigate the cryogenic mechanical properties of titanium alloys and reveal their deformation mechanisms [4,5,6,7,8].

For α titanium alloys, an ultra-high tensile strength (1200–1400 MPa) is obtained at 4–77 K [6] and there always are serrations in the stress-strain curves [5,9]. The critical shear stress to activate the dislocation glide systems of α-titanium alloys is thermally activated and the stress decreases greatly with the temperature increasing, while the stress to activate deformation twinning is not thermally activated [9]. Therefore, when compared with the dislocation glide, twining in α titanium alloys occurs more easily occur at a cryogenic temperature. For commercially pure titanium, high-density deformation twins could also be activated by the deformation at a cryogenic temperature [10]. The α + β type titanium, e.g., TC_4_, has a slightly higher tensile strength and a similar elongation than the α type titanium alloy [11]. Ambard et al. [11] found that, during the deformation at 20 K, there is only dislocation slip with very few twins in TC_4_. The deformation modes of TC_4_ are related to the morphology of α grains. In globular grains, the dominant system is the prismatic slip, but only the basal slip system is observed in lath colonies. With the addition of interstitial elements, the elongation of TC_4_ ELI increases to 18% and, meanwhile, the yield strength is as high as 1500 MPa [12]. Iorio et al. [12] claimed that twinning is the dominant deformation mechanism of TC_4_ ELI at 20 K and accounts for practically half of the plastic deformation.

In recent years, the properties of β-titanium alloys at cryogenic temperatures has drawn some attention [13,14,15,16,17]. Liu et al. reported that Ti-5Mo-5V-8Cr-3Al (TB2) had an ultra-high yield strength (1729 MPa) [13]. Saito et al. found that the elastic deformability of gum metal with cold swaging by 90% could exceed 4% at 77 K [14]. Wei et al. [15] investigated the elastic deformation behavior of gum metal at temperatures between 193 and 333 K and reported that the yield stress increased and the stress hysteresis became broader when decreasing the deformation temperature (233 to 193 K) for gum metal containing 0.3 wt.% oxygen. Gum metal also shows a low coefficient of a linear thermal expansion in a wide temperature region from 110 K to 613 K, which benefits its cryogenic practical applications [16]. Therefore, it could be deduced that β-titanium alloys have a prospect for application at cryogenic temperatures. However, there are relatively few research studies about the plastic deformation behavior of β-titanium alloys at cryogenic temperatures and the deformation mechanisms are not yet clear.

Our group has studied the plastic deformation behavior of gum metal with two different deformation histories at 77 and 200 K [17]. We found that both tensile strength and ductility of solution-treated gum metal have a significant enhancement at cryogenic temperatures. This study is a part of our ongoing research in the area of cryogenic plastic deformation behavior of β type titanium alloys. In this work, the Ti-36Nb-2Ta-3Zr-0.35O alloy with cold deformation by 58% was prepared. The mechanical properties and deformation mechanisms of the alloy at 77, 200, and 293 K were investigated to assess the possibility of its application at a cryogenic temperature. The cold deformation amount of the alloy (58%) falls in between those of the Ti-36Nb-2Ta-3Zr-0.35O alloy (0% and 85%) in our previous work [16] in order to investigate the effect of deformation history on the deformation behavior of the alloy.

## 2. Materials and Methods

The Ti-36Nb-2Ta-3Zr-0.35O (TNTZO) alloy used in this work was prepared by powder metallurgy and cold/hot deformation. The detailed preparation process has been described in our previous work [18]. Before sintering, the blended powders containing Ti, Nb, Ta, and Zr elemental powders were compacted by cold isostatic pressing (CIP) at a pressure of 200 MPa. In vacuum sintering, sintering temperature and sintering time were 1573 K and 16 h, respectively. Then the sintered bars were hot forged at temperatures ranging from 1073 to 1423 K and annealed at 1273 K for 1 h in argon, following cold swaging by 58% (reduction in cross section area, the sample named as EP0.58) at room temperature. In our previous work [16], the cryogenic mechanical properties of the as-annealed bars at 1273 K, which is named as EP0.0, and the bars after cold swaging by 85%, which is named as EP0.85, have been investigated.

Flat dog-bone-shaped specimens of a 9.5-mm gage length were used in tensile tests, which were carried out at a strain rate of 10^−4^ s^−1^and temperatures of 77, 200, and 273 K. At each temperature, three identical specimens were tested and every mechanical property value was averaged from these three independent tests. The following method was used to obtain the elongation to fracture, which has been used in our previous work [17]. Vickers micro-hardness indents spaced 1-mm apart were made along the specimen gage lengths before the tensile tests. The change in the distance between adjacent indents after the tensile tests was used to calculate the elongations to fracture.

The fracture surface was observed in a FEI Quanta FEG 250 scanning electron microscope (SEM, FEI, Hillsboro, OR, USA) operating at 15 kV. The microstructure of samples cut from the uniform deformation region after tensile tests was analyzed by a titan G2 60-300 transmission electron microscope (TEM, FEI, Hillsboro, OR, USA). TEM samples were prepared by a Twin-jet electro-chemical polishing technique and the etching solution contains 90% methanol (CH_3_OH) and 10% sulfuric acid (H_2_SO_4_).

## 3. Results and Discussions

**Figure 1:**
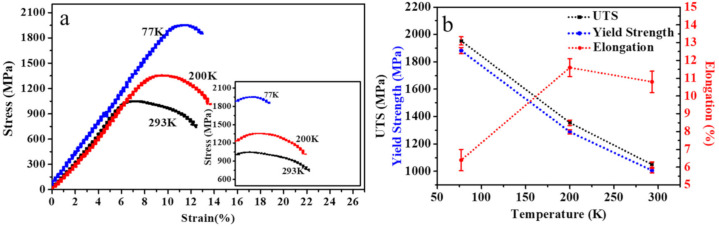
(**a**) Engineering stress-strain curves of EP0.58 (the insets in (**a**) is the stress-plastic strain curve), (**b**) yield strengths, ultimate tensile strengths (UTS), and elongations to fracture of EP0.58.

The engineering stress-strain curves of EP0.58 at different temperatures are shown in Figure 1a. Figure 1b summarizes yield strength, ultimate tensile strength, and elongation to fracture of EP0.58. The linear elastic portion is subtracted from the total stress-strain curve, and then only the plastic components are obtained (the insets in Figure 1a). In general, the flow stress increases and the engineering stress-strain curves shift up with the temperature decreasing from 293 K to 77 K, resulting in that yield and ultimate tensile strengths at cryogenic temperatures is much higher than the value of those at room temperature. Therefore, at 77 K, the yield strength and ultimate tensile strength reach the highest values of 1882 MPa and 1953 MPa, respectively. However, the elongation value for the alloy shows a complex temperature-dependent trend: increases first and then decreases with the decrease of temperature. The highest value (11.6%) of the elongation is obtained at 200 K. Thus, for the cold-swaged TNTZO alloy, the strength-ductility trade-off deviation occurs at 200 K.

The mechanical properties of EP0.0 [17], EP0.58, and EP0.85 [17] at 77 K, 200 K, and 293 K are summarized in Table 1. At room temperature, EP0.85 has the best comprehensive mechanical properties (yield strength: 1020 MPa and elongation: 14%). At 77 K, the elongation of EP0.0 increases to 20% and the strength is higher than 15 GPa. At 200 K, both the values of yield and ultimate strength of EP0.58 are the highest among those of TNTZO alloys with different cold deformations. Meanwhile, EP0.58 also maintains a proper elongation. Therefore, EP0.58 is more appliable at 200 K while EP0.0 is a preferred option for the application at 200 K.

**Figure 2:**
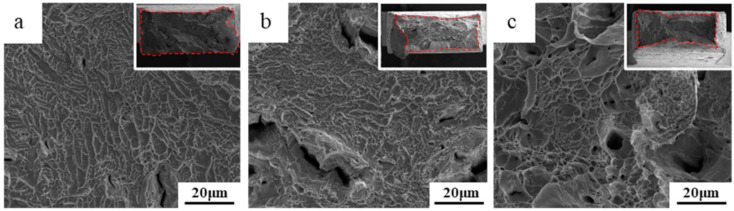
Fracture surfaces of EP0.58 at different temperatures: (**a**) 77 K, (**b**) 200 K, and (**c**) 293 K. The insets are the macrostructures of fracture surfaces.

The fracture surfaces of EP0.58 at different temperatures are shown in Figure 2. All of the tensile-fractured EP0.58 samples at different temperatures exhibit a dimple-like ductile fracture. With the temperature increasing, the average size of dimples in the fracture surfaces gradually becomes larger. Additionally, coarse dimples are observed clearly in the fracture surfaces of EP0.58 after tensile tests at room temperature (Figure 2c). The reduction of the area could be measured from the macrostructure of fracture surfaces, which are shown in the insets in Figure 2. All of the EP0.58 samples show plastic deformation under tensile stress at room and cryogenic temperature. EP0.58 after tensile tests at 293 K and 200 K have a similar area reduction (about 50%), which is much larger than that of the alloy at 77 K (about 15%).

**Figure 3:**
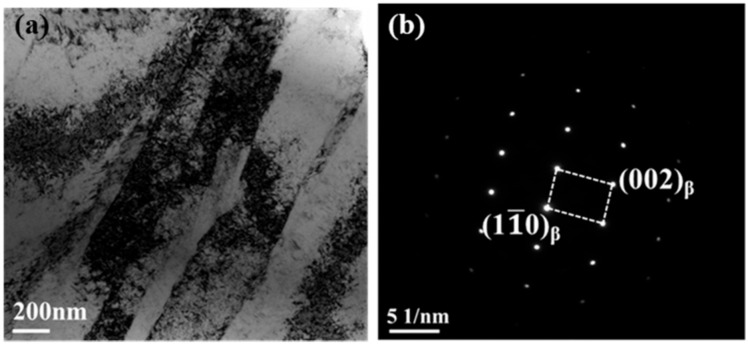
Microstructure of EP0.58 after tensile tests at 293 K: (**a**) Bright field image, (**b**) (110) β zone axis selected area electron diffraction (SEAD) patterns.

In order to shed some light on the strength-ductility trade-off deviation, the microstructure of the cold swaged alloy after tensile tests at different temperature (293, 200, and 77 K) is characterized by the Transmission Electron Microscope. Figure 3a shows the microstructure near the fracture of the cold swaged TNTZO alloy tested at room temperature and some micro shear bands are observed in which the width is slightly greater than that of “giant faults” detected in gum metal with a large deformation [19]. Only the electron-diffraction of the β-phase is detected in Figure 3b and there is no martensite and deformation twinning, indicating that the plastic deformation of the cold swaged alloy may be controlled by a dislocation motion.

**Figure 4:**
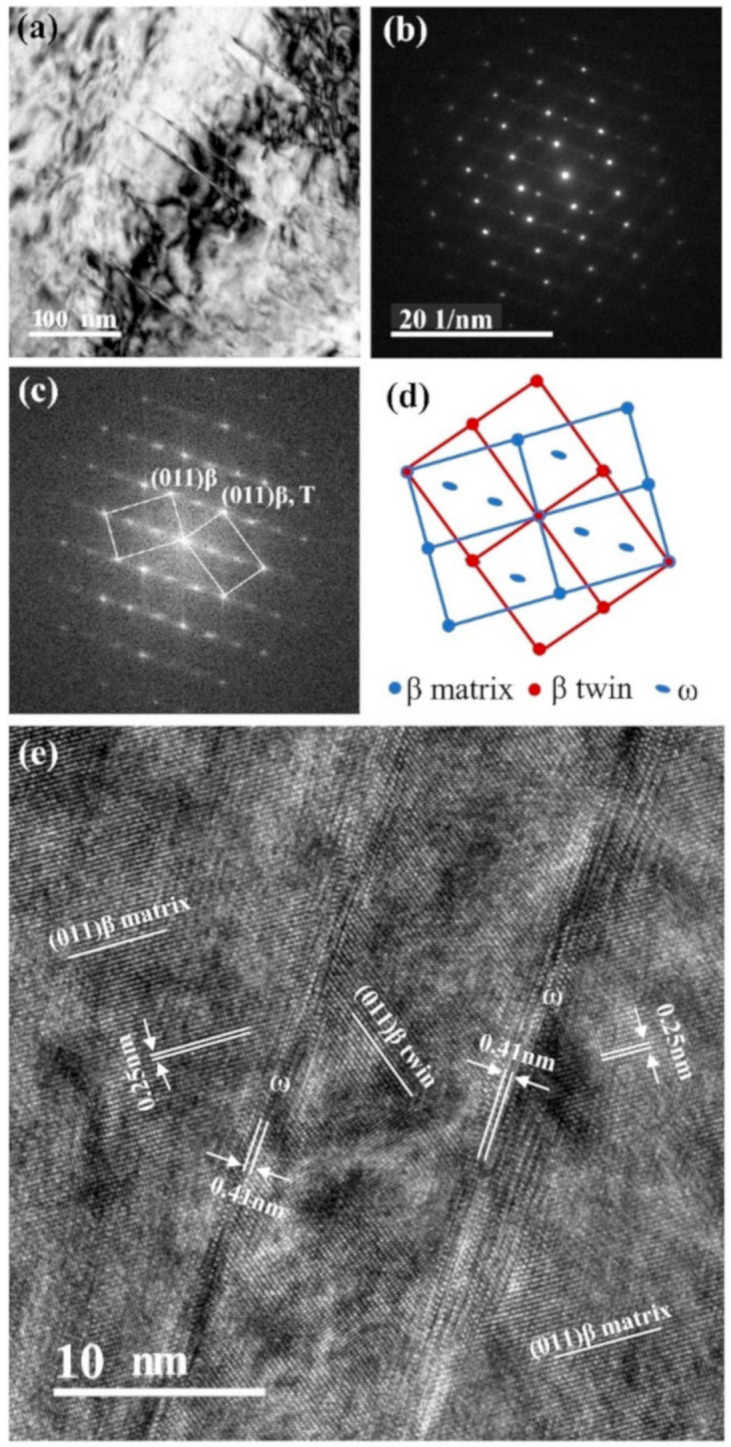
Microstructure of EP0.58 after tensile tests at 200 K: (**a**) Bright field image displaying several twin lamellae, (**b**) (110) β zone axis SAED patterns, (**c**) FFT patterns corresponding to the HRTEM image (**e**). (**d**) Key diagram corresponding to (**b**). (**e**) the HRTEM image of the twin lamellae in (**a**).

Figure 4 shows typical TEM images of the TNTZO alloy cold swaging by 58% after the tensile tests at 200 K. A certain amount of nano-sized lamellae are found in the bright-field image of the alloy (Figure 4a). The average width of these lamellae is about 7 nm, while their length is nearly 200 nm. The selected area electron diffraction (SAED) pattern (Figure 4b) and the corresponding key diagram (Figure 4d) indicate that they are {211} <111> twins. The diffuse ports of the ω phase, which appeared at the 1/3 position and 2/3 position can also be observed in Figure 4b and there are only two variants of the ω phase. A high resolution transmission electron microscopy (HRTEM) image (Figure 4e) shows that there is a layer of the ω phase along the boundary between {211} <111> twin and β matrix. The ω phase in this work is plate-like not particle-like, indicating that the ω phase is newly nucleated in the β phase [20]. The Fourier filtered transformation (FFT) image (Figure 4c) is consistent with the results obtained from the SEAD pattern and, in these two figures, the diffraction spots of the ω phase overlapped by the twinned β reflections is brighter. This is the first time that {211} <111> mechanical twinning accompanied with ω plate located at the twin boundaries is observed in the β titanium alloy after tensile tests at 200 K.

It has been proven that the ω plate along the {211} <111> twin boundary is introduced by the external stress nucleate on both the (211) composition plane of the mechanical twin. In addition, the shear along {211} <111> caused by the tensile stress is responsible for deformation twinning and ω transition [21]. Therefore, the ω phase is induced by {211} <111> twin at 200 K. In our previous work, the deformation twinning is also detected in the TNTZO alloy without cold deformation at 200 K, while the type of those twins is {332} <113> and no ω transition is found near the twins. It has been reported previously that the stress-induced β to ω phase transformation was also found to be accompanied with {332} <113> twinning in the titanium alloys with low β phase stability [22,23]. Therefore, it needs further study to explain why the different twinning systems with or without ω transition are observed during the tensile tests of the TNTZO alloy with a different cold deformation.

**Figure 5:**
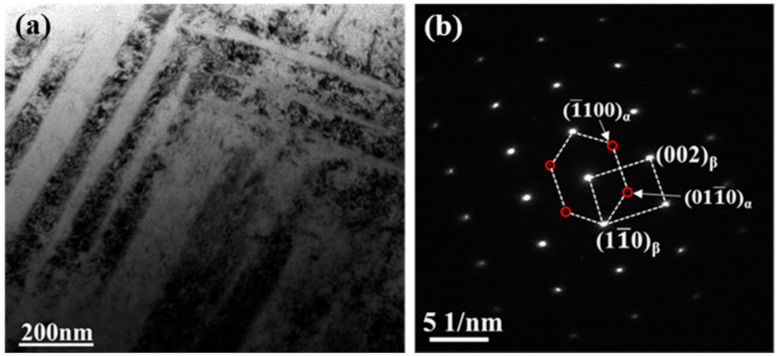
Microstructure of EP0.58 after tensile tests at 77 K: (**a**) Bright field image. (**b**) (110) β zone axis SEAD patterns.

Figure 5 shows the morphology of precipitates in the uniform deformation section of the tensile sample at 77 K. The plate-like precipitates are observed and the average width of the plates is about 80 nm. Figure 5b presents a selected area diffraction pattern, which shows clear extra spots corresponding to the α phase with the spots of the β phase, indicating that α phase precipitates in the β matrix during the tensile tests at 77 K. Li et al. [24] reported that, after deep cryogenic treatment for 12 h, the content of the α phase in TC_4_ was more than two times that of an untreated sample. In this work, the time of every tensile test at 77 K is no longer than 10 min, which is too short to precipitate α plates only owing to the cryogenic treatment. Therefore, it is then reasonable to conclude that the formation of the α phase is induced by the co-work of stress and cryogenic temperature.

The stability of the β phase is the dominant factor determining the deformation mechanisms of β titanium alloys and is always evaluated by the Bo-Md phase stability diagram [25,26,27,28]. The values of Bo and Md of titanium alloys are directly determined by alloy compositions [26,27]. In recent years, some research studies have proven that temperature also has an effect on the stability of the β phase [15,17,28]. For the TNTZO alloy, the β phase became more unstable with the temperature decreasing, resulting in martensitic transformation and twinning becoming easier to occur while slip becoming more difficult. Therefore, in this study, phase transformation and /or deformation twinning occur in the alloy during the tensile tests at 77 K and 200 K. We found that the stability of the β phase is sensitive to cold deformation history of the TNTZO alloy. At 200 K, only deformation twinning is observed in the TNTZO alloy without cold work and only ω phase precipitated in the β phase is detected in the TNTZO alloy with cold swaging by 85% [17]. However, both phenomena above are found in the TNTZO alloy with cold swaging by 58% in this work.

The trend of mechanical properties of EP0.58 with the temperature decreasing could be explained as follows. The formation of the plate-like ω phase along the {211} <111> twin boundary has been found in other titanium alloys with similar composition [20,21,29], while its effect on the mechanical properties is seldom reported. In this work, the enhancement of mechanical properties of the TNTZO alloy at 200 K is owing to this phenomenon and could be explained as follows. The formation of the ω phase always leads to a significant improved strength with a decreased elongation of the titanium alloy [30,31]. The formation of deformation twinning not only induces the strengthening but also has a twinning-induced plasticity (TWIP) effect [32,33]. Therefore, the increase in strength is due to the co-work of deformation twinning and the ω phase. Additionally, the elongation increased by the deformation twinning should be more than offset by the ω phase, resulting in a slight improvement of elongation of the TNTZO alloy with cold swaging by 58%. Many research studies have investigated the microstructural evolution of α phase during the heat treatment and deformation and its effect on the mechanical properties of titanium alloys [34,35,36,37]. These research studies have proven that the α phase in the metastable β titanium alloy is easy to induce poor ductility and high yield strength [34,36,38], which also account for the trend of strength and elongation of EP0.58 at 77 K.

## 4. Conclusions

In summary, the mechanical properties and deformation mechanism of the TNTZO alloy with cold swaging by 58% were investigated at different temperatures (from 77 K to 293 K). For the cold swaged alloy, a strength-ductility trade-off deviation is obtained at 200 K. During the tensile deformation at 200 K, the deformation twinning is formed, which also induces the ω phase to precipitate in the twin boundaries. The high tensile strength (1355 MPa) achieved at 200 K is owing to the fine-grained strengthening, resulting from deformation twinning and the precipitation strengthening of the hard ω phase. The good ductility (elongation: 11.6%) is due to the twinning induced plasticity effect. At 77 K, a significant increase of strength with a massive decrease of elongation is detected, which was mainly caused by martensitic transformation (the β phase to the α phase).

## Figures and Tables

**Table 1:** The values of ultimate tensile strength, yield strength, and elongation of the TNTZO alloys with different cold deformations [17].

Samples	Temperature (K)	UTS (MPa)	Yield Strength (MPa)	Elongation (%)
EP0.0	77	1569 ± 32	1213 ± 20	20.0 ± 2
EP0.58		1953 ± 30	1882 ± 23	6.4 ± 0.6
EP0.85		2040 ± 28	2008 ± 17	8.0 ± 0.8
EP0.0	200	1211 ± 18	1076 ± 15	14.9 ± 1.1
EP0.58		1355 ± 20	1288 ± 16	11.6 ± 0.5
EP0.85		1323 ± 17	1256 ± 20	10.0 ± 0.6
EP0.0	293	902 ± 15	860 ± 13	14.1 ± 1.2
EP0.58		1049 ± 20	1006 ± 17	10.8 ± 0.6
EP0.85		1078 ± 16	1020 ± 15	14.0 ± 0.9
